# Supplementary material for: Scope of health worker migration governance and its impact on emigration intentions among skilled health workers in Nigeria
Source: PLOS Glob Public Health. 2023 Jan 6;3(1):e0000717. doi: 10.1371/journal.pgph.0000717 (PMC10021292; doi:10.1371/journal.pgph.0000717)
Supplement: S1 File — (DOCX) [file pgph.0000717.s001.docx]

**S1 File: Steps taken for the Exploratory Factor Analysis**

| **Justification of the statistical analysis** |
| --- |
| To identify a relevant thematic area that captures the scope of health professional migration governance in Nigeria, we conducted an exploratory factor analysis (EFA) using maximum likelihood factor analysis.^(1, 2)^ Realising there was an unseen thinking pattern that informed participant’s’ choices during the survey, and hence response to an item was not completely independent of another, we chose EFA as the statistical approach for evaluating relationships between the survey items. In doing this we derived a smaller number of variables (latent factors) that accounted for the shared variance between the observable survey items.^(2)^ |
| **Adequacy of the extracted factors** |
| We had a sample size of 271 and with 38 core questions, this translated to 7:1 participant per item ratio and meets the Costello & Osborne’s recommendation.^(3)^ Since most of the survey items employed a Likert scale and had missing values, we did not assume normality of data. Hence, we used the function *corFiml* in the R package psych to compute a covariance matrix and submitted those to the EFA instead. To conduct the EFA, we used the *fa* function in the *psych* package in R. Bartlett’s test of sphericity was significant and confirmed that there was enough variability in the items to conduct an EFA (ᵪ2 = 3502.3, p <0.01, and df = 703). The Kaiser-Meyer-Olkin measure of sampling adequacy was 0.83, suggesting that it was acceptable to conduct an EFA. Evaluation of the factor correlation matrix showed correlation of between 0.1 and 0.48, suggesting a positive but weak correlation between the extracted factors. |
| To determine the number of factors to be extracted, we compared our findings from the Cartell's Scree plot Method (which suggested 8) and Horn’s Parallel analysis (which suggested three), and finally selected the eight-factor model based on what accounted for the most variability, and a conceptual interpretation of the extracted factors.^(4)^ We considered a survey item to be linked to a derived factor if it had a factor loading greater than 0.3 and had no secondary loadings (of greater than 0.3) on other factors. See Appendix 1 for how each survey item was liked to a derived factor. Six items (questions 7, 8, 11,17, 32, 37) had factor loadings less than 0.3, so were excluded. Question 36 cross-loaded on two factors so was also removed.^(5)^ For the retained items, communalities (i.e., the variance of each variable explained by its corresponding factors) ranged from 1.0 – 2.0. See Appendix 1. |
| **Rotation** |
| To describe the structural relationship between the survey items and the derived factors, we chose the Oblimin rotation method because it assumes correlation between items in a questionnaire. |
| **Scoring of the derived factors** |
| Using the scoreItems package in *R* ^(6)^, we then generated mean scores for each of the extracted factors and determined their reliability using Cronbach’s and Guttman’s Lambda 6 [G6(SMC)]. R statistical software was used for all the analyses. The final model included eight factors, with 31 items and explained 42% of the total survey variance. |

**References**

1. McNeish D. Exploratory Factor Analysis With Small Samples and Missing Data. Journal of Personality Assessment. 2017;99(6):637-52. doi:10.1080/00223891.2016.1252382

2. Sakaluk JK, Short SD. A Methodological Review of Exploratory Factor Analysis in Sexuality Research: Used Practices, Best Practices, and Data Analysis Resources. J Sex Res. 2017;54(1):1-9. doi:10.1080/00224499.2015.1137538

3. Costello AB, Osborne JW. Best practices in exploratory factor analysis: Four recommendations for getting the most from your analysis. Practical Assessment, Research & Evaluation. 2005;10:1-9. doi:10.7275/jyj1-4868

4. Izquierdo I, Olea J, Abad FJ. Exploratory factor analysis in validation studies: uses and recommendations. Psicothema. 2014;26(3):395-400. doi:10.7334/psicothema2013.349

5. Taber KS. The Use of Cronbach’s Alpha When Developing and Reporting Research Instruments in Science Education. Research in Science Education. 2017;48(6):1273-96. doi:10.1007/s11165-016-9602-2

6. R Core Team. R: A language and environment for statistical computing. R Foundation for Statistical Computing. Vienna, Austria; 2021.
