# Supplementary material for: Scope of health worker migration governance and its impact on emigration intentions among skilled health workers in Nigeria
Source: PLOS Glob Public Health. 2023 Jan 6;3(1):e0000717. doi: 10.1371/journal.pgph.0000717 (PMC10021292; doi:10.1371/journal.pgph.0000717)
Supplement: S2 File — (DOCX) [file pgph.0000717.s002.docx]

# **S2 File: Factor structure matrix**

|  | ML2 | ML5 | ML1 | ML3 | ML4 | ML8 | ML6 | ML7 | h2 | u2 | com |
| --- | --- | --- | --- | --- | --- | --- | --- | --- | --- | --- | --- |
| q1 | 0.11 | -0.02 | 0.01 | -0.02 | -0.08 | -0.01 | 0.01 | **0.67** | 0.47 | 0.53 | 1.1 |
| q2 | -0.05 | -0.12 | 0.10 | 0.03 | 0.07 | -0.08 | -0.02 | **0.61** | 0.40 | 0.6 | 1.2 |
| q7 | -0.20 | 0.13 | -0.03 | 0.02 | 0.13 | 0.17 | 0.05 | 0.19 | 0.17 | 0.83 | 4.8 |
| q32 | 0.02 | 0.02 | 0.03 | -0.10 | 0.12 | -0.24 | 0.22 | -0.04 | 0.13 | 0.87 | 3 |
| q3 | -0.08 | 0.24 | -0.03 | -0.01 | 0.04 | 0.05 | 0.17 | **0.39** | 0.29 | 0.71 | 2.2 |
| q4 | -0.02 | 0.07 | -0.04 | -0.06 | 0.23 | **0.40** | 0.14 | -0.08 | 0.30 | 0.7 | 2.2 |
| q5 | -0.05 | 0.00 | -0.01 | 0.02 | 0.04 | **0.68** | -0.03 | -0.02 | 0.48 | 0.52 | 1 |
| q6 | 0.04 | -0.06 | 0.09 | 0.01 | -0.04 | **0.70** | 0.04 | -0.01 | 0.50 | 0.5 | 1.1 |
| q8 | -0.04 | 0.19 | -0.05 | -0.01 | 0.17 | 0.23 | 0.26 | 0.16 | 0.33 | 0.67 | 4.5 |
| q9 | -0.04 | 0.07 | 0.01 | -0.03 | 0.12 | 0.02 | 0.09 | **0.33** | 0.17 | 0.84 | 1.6 |
| q10 | 0.03 | 0.02 | 0.01 | -0.05 | **0.45** | 0.17 | 0.08 | 0.03 | 0.32 | 0.68 | 1.4 |
| q11 | -0.08 | 0.06 | 0.08 | 0.10 | -0.02 | -0.07 | 0.10 | -0.06 | 0.04 | 0.96 | 6.3 |
| q12 | 0.17 | 0.15 | 0.12 | -0.05 | **0.35** | 0.03 | 0.03 | -0.03 | 0.32 | 0.69 | 2.3 |
| q13 | 0.13 | 0.08 | 0.12 | -0.07 | **0.51** | 0.12 | -0.05 | 0.12 | 0.46 | 0.54 | 1.6 |
| q14 | **0.36** | -0.09 | 0.02 | 0.02 | 0.19 | 0.03 | 0.00 | -0.11 | 0.19 | 0.81 | 1.9 |
| q15 | 0.06 | 0.15 | 0.01 | 0.12 | **0.47** | -0.13 | 0.06 | 0.04 | 0.38 | 0.62 | 1.6 |
| q16 | -0.12 | 0.23 | 0.17 | 0.08 | **0.53** | 0.10 | 0.10 | -0.03 | 0.66 | 0.34 | 1.9 |
| q17 | 0.04 | 0.01 | 0.00 | -0.03 | 0.21 | -0.10 | -0.02 | 0.22 | 0.11 | 0.89 | 2.5 |
| q18 | 0.26 | -0.13 | -0.03 | 0.07 | **0.45** | -0.04 | 0.10 | 0.00 | 0.34 | 0.66 | 2 |
| q19 | 0.05 | 0.07 | **0.62** | -0.01 | 0.25 | 0.01 | -0.04 | 0.05 | 0.62 | 0.38 | 1.4 |
| q20 | 0.01 | 0.00 | **0.92** | 0.01 | -0.03 | -0.05 | 0.02 | 0.01 | 0.83 | 0.17 | 1 |
| q21 | -0.02 | 0.06 | **0.69** | 0.02 | -0.06 | 0.17 | 0.02 | 0.02 | 0.59 | 0.41 | 1.2 |
| q22 | 0.01 | -0.03 | 0.15 | **0.65** | -0.02 | 0.00 | 0.10 | -0.06 | 0.52 | 0.48 | 1.2 |
| q23 | -0.05 | 0.02 | -0.03 | **0.79** | 0.07 | 0.00 | 0.05 | 0.03 | 0.65 | 0.35 | 1 |
| q24 | 0.27 | -0.20 | 0.03 | **0.33** | 0.22 | -0.05 | 0.04 | -0.02 | 0.30 | 0.7 | 3.6 |
| q25 | 0.12 | 0.14 | -0.05 | **0.62** | -0.15 | 0.03 | -0.08 | 0.02 | 0.44 | 0.56 | 1.4 |
| q26 | 0.03 | 0.04 | -0.03 | -0.02 | -0.04 | -0.02 | **0.79** | -0.01 | 0.62 | 0.38 | 1 |
| q27 | 0.03 | -0.06 | 0.06 | 0.11 | 0.02 | 0.05 | **0.69** | 0.04 | 0.58 | 0.42 | 1.1 |
| q28 | **0.69** | 0.14 | 0.01 | 0.00 | -0.05 | -0.11 | 0.02 | 0.06 | 0.56 | 0.44 | 1.2 |
| q29 | **0.68** | 0.00 | 0.01 | -0.10 | 0.07 | -0.05 | 0.13 | -0.01 | 0.56 | 0.44 | 1.2 |
| q30 | **0.54** | 0.03 | -0.08 | 0.13 | 0.12 | 0.17 | -0.06 | 0.12 | 0.42 | 0.58 | 1.6 |
| q31 | **0.66** | -0.04 | 0.09 | 0.13 | -0.03 | 0.09 | 0.05 | 0.01 | 0.54 | 0.46 | 1.2 |
| q33 | 0.00 | **0.50** | 0.10 | 0.19 | 0.13 | 0.01 | 0.02 | -0.04 | 0.48 | 0.52 | 1.5 |
| q34 | 0.00 | **0.61** | 0.13 | 0.06 | -0.01 | -0.01 | 0.05 | 0.06 | 0.53 | 0.47 | 1.2 |
| q35 | 0.06 | **0.72** | 0.06 | 0.02 | 0.08 | -0.05 | 0.04 | -0.05 | 0.66 | 0.34 | 1.1 |
| q36 | **0.32** | **0.41** | 0.09 | -0.05 | -0.02 | -0.11 | 0.15 | -0.09 | 0.44 | 0.56 | 2.6 |
| q37 | 0.00 | 0.28 | -0.02 | 0.17 | 0.00 | 0.26 | -0.08 | 0.11 | 0.24 | 0.76 | 3.2 |
| q38 | 0.03 | **0.47** | 0.07 | -0.01 | 0.02 | 0.29 | 0.02 | 0.07 | 0.44 | 0.56 | 1.8 |

**Key: (1)** Items with a yellow background, loaded appropriately on the respective factors, those with grey had factor loadings less than 0.3, while Q 36 cross loaded on two factors above 0.3. **(2)** h2 = communality, u2 = residual variance (uniqueness), com = Hoffman’s index of complexity.
