# Supplementary material for: Scope of health worker migration governance and its impact on emigration intentions among skilled health workers in Nigeria
Source: PLOS Glob Public Health. 2023 Jan 6;3(1):e0000717. doi: 10.1371/journal.pgph.0000717 (PMC10021292; doi:10.1371/journal.pgph.0000717)
Supplement: S3 File — (DOCX) [file pgph.0000717.s003.docx]

# **S3 File: Factor correlation matrix**

|  | | | | | | | | |
| --- | --- | --- | --- | --- | --- | --- | --- | --- |
|  | **F2** | **F5** | **F1** | **F3** | **F4** | **F8** | **F6** | **F7** |
| F2 | 1 | 0.19 | 0.19 | 0.26 | 0.23 | -0.1 | 0.36 | 0.14 |
| F5 | 0.19 | 1 | 0.48 | 0.21 | 0.34 | 0.18 | 0.35 | 0.1 |
| F1 | 0.19 | 0.48 | 1 | 0.23 | 0.34 | 0.24 | 0.32 | 0.22 |
| F3 | 0.26 | 0.21 | 0.23 | 1 | 0.14 | 0.19 | 0.27 | 0.08 |
| F4 | 0.23 | 0.34 | 0.34 | 0.14 | 1 | 0.24 | 0.32 | 0.13 |
| F8 | -0.1 | 0.18 | 0.24 | 0.19 | 0.24 | 1 | 0.09 | 0.12 |
| F6 | 0.36 | 0.35 | 0.32 | 0.27 | 0.32 | 0.09 | 1 | 0.09 |
| F7 | 0.14 | 0.1 | 0.22 | 0.08 | 0.13 | 0.12 | 0.09 | 1 |

**Key for the Factors:** (1) Government’s efforts towards political, and economic stability,

(2) Unilateral/cooperative approaches to governance, (3) Efforts by non-state actors,

(4) International and national health workforce policies, (5) Government and citizen’s commitment to human rights broadly, and the RTH specifically, (6) Efforts by patients and community groups to support SHWs, (7) SHW’s personal utility in choosing to migrate, and (8) government’s commitment to improving working conditions and remunerations for SHWs.
