# Supplementary material for: Scope of health worker migration governance and its impact on emigration intentions among skilled health workers in Nigeria
Source: PLOS Glob Public Health. 2023 Jan 6;3(1):e0000717. doi: 10.1371/journal.pgph.0000717 (PMC10021292; doi:10.1371/journal.pgph.0000717)
Supplement: S4 File — (DOCX) [file pgph.0000717.s004.docx]

**S4 File: Variance explained by an eight-factor model**

|  | | | | | | | | | |  |
| --- | --- | --- | --- | --- | --- | --- | --- | --- | --- | --- |
|  | F2 | F5 | F1 | F3 | F4 | F8 | F6 | F7 |  |  |
| SS loadings | 2.45 | 2.46 | 2.25 | 1.89 | 2.17 | 1.77 | 1.7 | 1.38 |  |  |
| Proportion Var | 0.06 | 0.06 | 0.06 | 0.05 | 0.06 | 0.05 | 0.04 | 0.04 |  |  |
| Cumulative Var | 0.06 | 0.13 | 0.19 | 0.24 | 0.3 | 0.34 | 0.39 | *0.42 |  |  |
| Proportion Explained | 0.15 | 0.15 | 0.14 | 0.12 | 0.14 | 0.11 | 0.11 | 0.09 |  |  |
| Cumulative Proportion | 0.15 | 0.31 | 0.45 | 0.56 | 0.7 | 0.81 | 0.91 | 1 |  |  |

**Key for the Factors:** (1) Government’s efforts towards political, and economic stability,

(2) Collaborative approaches to governance, (3) Efforts by non-state actors,

(4) International and national health workforce policies, (5) Commitment to human rights norms, (6) Efforts by patients and community groups to support SHWs, (7) SHW’s perceived benefit of remaining in Nigeria , and (8) government’s commitment to improving working conditions and remunerations for SHWs.

**Var** = variance, SS = rotated sum of square loadings, **F** = Factors. *Total variance accounted for by the retained items.
