# Supplementary material for: Scope of health worker migration governance and its impact on emigration intentions among skilled health workers in Nigeria
Source: PLOS Glob Public Health. 2023 Jan 6;3(1):e0000717. doi: 10.1371/journal.pgph.0000717 (PMC10021292; doi:10.1371/journal.pgph.0000717)
Supplement: S5 File — (DOCX) [file pgph.0000717.s005.docx]

# **S5 File: Factor labels and their corresponding survey questions**

| **Factors** | **Survey item** | | **Factor Loading** | **Factor Label** |
| --- | --- | --- | --- | --- |
| Factor 1 | q19 | There are progressive efforts by the Nigerian government to improve economic prosperity and reduce inflation. | 0.62 | Government’s efforts towards political-and economic stability |
|  | q20 | There have been progressive efforts by the Nigerian government towards improving political stability. | 0.92 |  |
|  | q21 | There have been progressive efforts by the Nigerian government towards controlling corruption. | 0.69 |  |
| Factor 2 | q14 | The Nigerian government has taken steps (e.g., the use of bonds, ban on traveling) for preventing migration of health professionals. | 0.36 | Collaborative approaches to SHW migration governance (by the government and other stakeholders within and outside the country) |
|  | q28 | There is a government agency responsible for the design and implementation of rules related to skilled health worker migration in Nigeria. | 0.69 |  |
|  | q29 | Government agencies and non-governmental parties (e.g., patient groups, the private sector, civil society organizations, etc.) work together to design and implement rules guiding emigration of skilled health workers from Nigeria. | 0.68 |  |
|  | q30 | Nigeria is a signatory to bilateral agreements aimed at reducing the negative impact of skilled health worker emigration. | 0.54 |  |
|  | q31 | Health professional organizations regularly monitor the trends for emigration of health professionals from Nigeria and have rules for ensuring it does not affect the country's health workforce needs. | 0.66 |  |
| Factor 3 | Q22 | Civil Society Organizations in Nigeria regularly promote awareness for skilled health worker shortages in the country. | 0.65 | Efforts by civil society organisations and health professional groups |
|  | Q23 | Health professional organizations in Nigeria regularly promote awareness for skilled health worker shortages in the country. | 0.79 |  |
|  | Q24 | Health professional organizations in Nigeria are taking steps to discourage skilled health workers from migrating out of the country. | 0.33 |  |
|  | q25 | I am involved in efforts aimed at increasing awareness of skilled health worker shortages in my community. | 0.62 |  |
| Factor 4 | q10 | Communities in Nigeria are gaining more access to the skilled health services they need. | 0.45 | Health Workforce Policies |
|  | q12. | Nigeria has a strategy for encouraging the return of skilled health workers that have migrated. | 0.35 |  |
|  | q13 | In Nigeria, the government (at various levels) has increased recruitment of health professionals. | 0.51 |  |
|  | q15 | There are efforts aimed at encouraging skilled health workers who have emigrated, to invest in Nigeria. | 0.47 |  |
|  | q16 | There are ongoing efforts by the Nigerian government to improve work conditions at all health institutions (e.g., medical colleges, hospitals) in the country | 0.53 |  |
|  | q18 | The World Health Organization (WHO) Code for the ethical recruitment of international health professionals has inspired the Nigerian government's response to skilled health worker migration. | 0.45 |  |
| Factor 5 | Q33 | The right of everyone to have the highest attainable standard of health is recognized by the government of Nigeria. | 0.50 | Commitment to human right norms (including the Right to Health & SHW’s right to gainful employment) |
|  | Q34 | There is a commitment by the Nigerian government towards non-discrimination (e.g., non-discrimination based on gender, ethnic origin, race, or colour; age; disability; language; religion; national origin; socioeconomic or political status). | 0.61 |  |
|  | Q35 | In Nigeria, there are mechanisms to ensure that the government protects its citizens' right to health | 0.72 |  |
|  | Q38 | Health professionals’ right to work and earn fair wages in Nigeria, is respected by the government | 0.47 |  |
| Factor 6 | q26 | I feel valued because my patients appreciate the services I offer. | 0.79 | Support by patients and community groups |
|  | q27 | I feel supported by the community served by my hospital/clinic. | 0.69 |  |
| Factor 7 | Q1 | If I practice abroad, I can earn a higher salary and offer my family better access to health care, education, and safety. | 0.67 | Perceived utility of remaining in Nigeria |
|  | Q2 | If I practice abroad, I will acquire further professional and technical skills. | 0.61 |  |
|  | Q3 | In Nigeria, opportunities for advancing my career and specialization are available and accessible | 0.39 |  |
|  | Q9 | I do not wish to deal with the cost of emigration (i.e., re-qualification, going through an immigration process, uncertainties, and the time required to learn a new system). | 0.33 |  |
| Factor 8 | q4 | I am satisfied with the way health services and work conditions are managed at my hospital/clinic. | 0.40 | SHW’s satisfaction with working conditions and remunerations |
|  | q5 | I am satisfied with the regularity of payment of salaries at my organization | 0.68 |  |
|  | q6 | I am satisfied with how my salary compares with that of colleagues with the same qualification, working at other levels of health care in Nigeria. | 0.70 |  |
