# Supplementary material for: Scope of health worker migration governance and its impact on emigration intentions among skilled health workers in Nigeria
Source: PLOS Glob Public Health. 2023 Jan 6;3(1):e0000717. doi: 10.1371/journal.pgph.0000717 (PMC10021292; doi:10.1371/journal.pgph.0000717)
Supplement: S6 File — (DOCX) [file pgph.0000717.s006.docx]

# **S6 File: Thematic index of the qualitative findings**

|  | Operational Level | Collective Level | Constitutional Level |
| --- | --- | --- | --- |
| Item | **Theme/sub-themes** | **Theme/ sub-themes** | **Theme/ sub-themes** |
| Governing rules/norms | **SHWs’ involvement in health advocacy and engagement**   - Influencing colleagues’ migration intentions - Creating awareness for the RTH among patients & colleagues - Engaging with policy makers on improving health services   **SHWs’ role in ensuring access to health services**   - Responsibility of the SHW to provide quality health services | **Social and financial support for SHW migration**   - Recruitments and financial support through private agencies - Support/expectations from family members   **Advocacy and engagement by non-state actors**   - Advocacy and engagements by health professional groups, journalists, civil society organisations, and non-governmental organisations - Limited inputs by the broader society   **Oversight by hospital administrators**   - Approval for international travels - Working conditions   **Health worker resilience, interactions, and advocacy**   - Adaptation to challenges, - Inter/intra professional relationships | - **Funding the health system** - **Managing the skilled health workforce** - **SHW migration** |
| Factors influencing accountability to governing rules/norms | **Attitude towards migration**   - SHW’s preference for temporary migration - Migration, an acceptable option for addressing unemployment and receiving foreign remittances   **Commitment towards building the health system**   - Full commitment to building the Nigerian health system - Partial commitment while seeking migration opportunities - Discouraged about the impact of their contribution   **Finding personal fulfilment**   - Interacting with contacts abroad while practicing in Nigeria - Service to humanity, not just Nigerians   **Attitude towards the right to health**   - Recognises a social contract with patients - Appreciates the need for collective action - The right to health as important as SHWs’ right to migrate | **Attitudes of stakeholders outside the health system**   - Citizens’ poor perception of SHWs - Lack of awareness of the right to health within the society - Mixed attitude towards SHW migration within the society   **Attitude of stakeholders within the health system**   - Nonchalant attitude to migration by health practitioner regulatory agencies - SHW’s moral dilemma with migration - Nonchalant attitude to the RTH or the medical profession among health system leaders - SHWs subscribe to the RTH but lack commitment to its implementation - SHWs’ are not aware of legal tools for upholding the RTH | **Attitude towards SHW migration**   - Not recognised as a problem - Supplies remittances to support the national economy - Migration provides gainful employment for SHWs, absolves the government of further responsibility.   **Attitude towards the right to health**   - A lack of care for the citizens’ wellbeing - Government’s focus on hospital services, ignoring disease prevention & health promotion |
| Outcome | **Personal satisfaction that comes from improved patient outcomes** | **Patients’ experience with the health system**   - Long hospital waiting times - Preference for traditional over hospital care   **Good and poor health worker training outcomes**  **Skilled health workers’ practice experience**   - Insufficient manpower - Health worker exhaustion - Inadequate quality of health services | - **A lack of universal health coverage.** |
