# Supplementary material for: Scope of health worker migration governance and its impact on emigration intentions among skilled health workers in Nigeria
Source: PLOS Glob Public Health. 2023 Jan 6;3(1):e0000717. doi: 10.1371/journal.pgph.0000717 (PMC10021292; doi:10.1371/journal.pgph.0000717)
Supplement: S7 File — (DOCX) [file pgph.0000717.s007.docx]

# **S7 File: Mapping the quantitative results to the qualitative findings**

| Quantitative findings | Qualitative findings |
| --- | --- |
| Factor 1 - had a low median score of 1.67 suggesting that government’s efforts towards ensuring political, economic, and social stability was poor | Even though it is not captured as a governing rule/norm in the qualitative findings, this score aligns with the participant’s perceptions about the government’s inability to address challenges with the economy. They thought the government relied on remittances the country receives from SHWs who migrate to support the economy (see governing norms for SHW migration at the constitutional level. |
| Factor 2 has a median score of 2.4 reflecting poor collaboration between stakeholders in the governance of SHW migration | This aligns with the qualitative findings reflecting little collaboration across the three levels of governance |
| Factor 3 has a median score of 3.0, reflecting a neutral perspective on efforts by non-state actors (health professional groups and civil society actors) towards increasing awareness of SHW shortages and encouraging the retention of SHWs was perceived by the participants. | Even though the qualitative findings (in the section on collective level governing rules/norms above) clearly captured the efforts of health professional groups and civil society actors, the participants also mentioned that a lot more could be done. In addition, the influence of private recruiters, financial agents, and family members encouraging migration may have overshadowed advocacy efforts on addressing SHW shortages |
| Factor 4 has a median score of 2.0 suggests a poor perspective of government’s health workforce policies | The qualitative findings on the other hand are clear that recruitment and remuneration have been poor. Similarly, both national and international efforts for handling SHW migration have been sub-optimal |
| Factor 5 has a median score of 2.25 denoting a low level of commitment by government and citizens towards human right norms | A few of the participants shared a positive attitude towards the RTH. However, they also noted a poor attitude to the human rights norms across the various levels of governance |
| Factor 6 has a median score of 3.5 suggesting positive perspective of SHW support by patients and communities in Nigeria. | This was not captured in the qualitative findings as a distinct governing rule/norm. |
| Factor 7 has a low median score of 2.00 suggesting that the SHWs considered more benefit in traveling compared to remaining. | The qualitative findings suggest a tension between SHW’s personal utility, commitment to the RTH, right to migrate, and remaining to build the health system |
| Factor 8 with a low median score of 2.00 represents SHWs’ poor satisfaction with the government’s efforts towards improving working conditions and remuneration. | The qualitative findings (especially the sub theme on *training and remuneration of SHWs* above) suggests the same |
