# Supplementary material for: Scope of health worker migration governance and its impact on emigration intentions among skilled health workers in Nigeria
Source: PLOS Glob Public Health. 2023 Jan 6;3(1):e0000717. doi: 10.1371/journal.pgph.0000717 (PMC10021292; doi:10.1371/journal.pgph.0000717)
Supplement: Attachment — Submitted filename: Response to Reviewers.docx [file pgph.0000717.s008.docx]

**Journal Requirements:**

1. Please amend your online Financial Disclosure statement. If you did not receive any funding for this study, please simply state: “The authors received no specific funding for this work.”

**Authors response:** We have stated this on the online financial disclosure statement

2. Please update your online Competing Interests statement. If you have no competing interests to declare, please state: “The authors have declared that no competing interests exist.”

**Authors response:** We have stated this on the online competing interests statement

3. We do not publish any copyright or trademark symbols that usually accompany proprietary names, eg (R), (C), or TM  (e.g. next to drug or reagent names). Please remove all instances of trademark/copyright symbols throughout the text, including © (SurveyMonkey©) on page 7.

**Authors response:** We have done this

4. We have noticed that you have uploaded Supporting Information files, but you have not included a list of legends. Please add a full list of legends for your Supporting Information files after the references list.

**Authors response:** We have done this on page 40, lines 929 - 940

5. Please review your reference list to ensure that it is complete and correct. If you have cited papers that have been retracted, please include the rationale for doing so in the manuscript text, or remove these references and replace them with relevant current references. Any changes to the reference list should be mentioned in the rebuttal letter that accompanies your revised manuscript. If you need to cite a retracted article, indicate the article’s retracted status in the References list and also include a citation and full reference for the retraction notice.

********************************************************************************

**Reviewer #1: This is an interesting paper, one that can contribute to the literature on SHW migration. I have a few suggestions to strengthen the paper**

**1. I appreciate the focus on the rights-based migration approach, but you might also want to relate this what some authors call 'whole of government or whole of society' approach, for example see Buchan, J., Campbell, J., Dhillon, I., & Charlesworth, A. (2019). Labour market change and the international mobility of health workers. Health Foundation working paper, 5.**

Authors’ response: We thank the reviewer for this comment. We agree that a whole of government approach is necessary for responding to the challenges associated with skilled health worker migration. We previously used the term multi-sectoral approach to capture this but have now changed this to whole of government/society approach.

On page 27 as from line 609 we stated this as follows: “Such stakeholder engagements can lead to innovative application of the WHO Code and other initiatives (including a **whole of government/society approach**) useful for mitigating the negative impact of SHW migration ^(55, 56, 68)^,

and on page 30, lines 679 – 682 we stated the following: “Adopting a rights-based approach to SHW migration governance will require that the government recognise the need for a **whole of government/society approach**, seek to engage more with foreign governments, and invite citizens to hold it accountable to an agreed set of processes and outcomes.”.

We also explained how our analytical approach affirms the whole of government/society approach on page 28, line 613 – 617: “In adopting an institutional approach, we affirm existing recommendations **for a whole of government/society approach** for handling SHW migration ^(69)^ and the implications this has for setting up relevant governance structures (“hardware”). Our approach also recognises the need for relational (“soft-ware”) aspects that should be considered in understanding the scope of SHW migration governance ^(70, 71)^.”

**2. I appreciate the multilevel analysis of the data, but you are missing a fourth level, that of the international or global level. You mention the WHO Code, which would be relevant to this scale, but also relevant are bilateral agreements, regional mobility agreements, international conventions that apply (even if they are ignored). You do mention some of these agreements, but you have not included this level in your model. It is relevant even if that relevance is due to public and state reluctance to engage with it.**

Authors’ response: We thank the reviewer for this comment and agree that an international dimension to SHW migration governance is important. We have included this in the current framework. Please see figure 1. Since our interest is the scope of SHW migration governance in Nigeria, we have kept it within the “constitutional level” but ensured that our audience appreciate the link to regional or international health workforce conventions.

On page 26 as from line 575, we have reflected on our findings that suggest a low level of engagement with the WHO Code in Nigeria as follows: “Promoting the right to health often reveals a tension with the right to migrate. The **WHO Code** seeks to address this tension by promoting ethical recruitment of SHWs, bilateral/multilateral agreement on health workforce migration, and domestic efforts towards achieving a sustainable health workforce. Drawing from experiences in Sudan, the **WHO Code** can help trigger public discourse on SHW migration and bilateral agreements on its regulation in Nigeria ^(59)^. However, our study suggests a low awareness of the **WHO Code** or its role in the governance of SHW migration in Nigeria, consistent with findings from a study exploring its relevance in eastern and southern Africa ^(60)^”

**3. Under 'material and methods' the section on sample size needs to be more clearly explained, including providing the actual number of surveys and interviews conducted versus planned, and the relationship of the survey results to the interview script. Also a table might be useful to show the different methods used and how they related to each other (what order they occurred in and how one influenced or not the other). The literature review is also a method, and so more information on how this was conducted and how it informed other methods would be useful.**

Authors’ response: We have stated this on page 6, lines 137-139 and lines 140-144; our **estimation was a minimum sample size of 250** but the actual number of people **we eventually recruited to participate in the survey was 271**.

For the interviews, we estimated we will be able to **interview 20 participants**, and we eventually interviewed **22**.

In figure 2, we provided an overview of the different methods used in this study.

**4. The discussion on the balance between right to health and right to migrate is also key to the WHO Code, which indicates SHW right to migration but also in light of their legal responsibilities. You could discuss the Code in more detail, particularly if you include it as one of the levels of analysis. Also your comments on the Code drawn from the interviews needs to be more contextualized. You need to briefly explain the code and its relevance to Nigeria (Nigeria is not on the safeguard list, but is a member of WHA so must have approved the Code in 2010). Since your focus is on governance the global governance aspect of this phenomena should be considered.**

Authors’ response: We thank the reviewer for this comment and agree on the need to contextualise the WHO Code. We have done this on page 26, line 575-585 as follows:

Promoting the right to health often reveals a tension with SHWs’ right to migrate. The **WHO Code** seeks to address this tension by promoting ethical recruitment of SHWs, bilateral/multilateral agreement on health workforce migration, and domestic efforts towards achieving a sustainable health workforce. Drawing from experiences in Sudan, the **WHO Code** can help trigger public discourse on SHW migration and bilateral agreements on its regulation in Nigeria ^(60)^. However, our study suggests a low awareness of the **WHO Code** or its role in the governance of SHW migration in Nigeria, consistent with findings from a study exploring its relevance in eastern and southern Africa ^(61)^. Even if poor engagement with the **WHO Code** can be explained by state actors’ disagreement with its relevance in an African context ^(61)^, the absence of civic engagement on SHW migration and its impact on population health suggests a more fundamental issue regarding how this issue is perceived in Nigeria.

**5. You mention on p28 that this approach is a useful tool of analysis to explore SHW in Nigeria, but you should also consider how it can be used in other national settings. You should be more assertive in the contribution your approach can make to the literature on SHW migration.**

Authors’ response: We thank the reviewer for this comment. We have modified this accordingly on page 28 line 632-640 to reflect its utility for other national settings:

“As with all complex, wicked problems, a frame of reference is needed to guide a country’s response to SHW migration ^(77)^. Beyond an understanding of the scope of skilled health migration governance in Nigeria, our framework provides multiple entry points and interconnections useful for understanding how SHW migration is handled in under governed settings. It thus offers insights for designing future policy interventions and evaluating their utility for achieving health workforce sustainability in Nigeria and other national settings facing similar challenges.”

********************************************************************************

**Reviewer #2: This is article focuses on the governance of health worker migration as perceived by health workers themselves. I am not especially convinced by the methodology and methods used, but I recognise that there are other views in the field and that a post-structuralist 'governmentality' of health worker migration might, for many colleagues, be a viable approach. Nevertheless, the authors frame this in terms of governance, by which I think they probably mean governance, regulation and policy, which is different from governmentality. The paper is well-written and -presented. The data on health worker (e)migration (including return migration) could meaningfully and usefully be updated (presently it stops at 2011 - more than 10 years ago!).**

**Suggested revisions:**

**1. Clarify and justify the choice of governance as an analytical device (as opposed to regulation, policy, law and governmentality).**

We thank the reviewer for the invitation to clarify and justify our choice of governance as an analytical device. We have done this on page 28, line 618-626 as follows: “By eliciting the perceptions of our participants, we align with an interpretivist approach to governance; one that explains the governance through actions, explores belief and meaning for an in-depth understanding of these actions, recognises governance as including top-down and bottom-up practices, and explores its contingent nature (that is, governance as having no definite causal path) ^(12, 72)^. Underpinning these actions is an interplay of power. Hence, we recognise that a post-structuralist governmentality of health worker migration offers an alternative analytical framework ^(73)^. However, in Nigeria (as in many other LMICs), there is a tendency for state actors to under govern. Hence, our preference for governance as an analytical tool, specifically, a structure-relational approach ^(12, 74)^.”

**2. Clarify and justify the decision to focus on subjective understandings of individual migrant health workers viz governance and what new perspectives this potentially brings.**

We thank the reviewer for this comment and have responded on page 28/29, line 627-638 as follows: “The subjective judgements of our participants are relevant to a specific context, and thus, cannot be generalised. However, we do not consider this to be a limitation to the usefulness of our framework. SHWs are the primary targets of any formal policy/regulation on migration. Their perception and response to existing rules (or a lack of rules) drives migration behaviour ^(76, 77)^. In addition, their reasoning can be influenced by interactions (or lack thereof) with other relevant stakeholders. As with all complex, wicked problems, a frame of reference is needed to guide a country’s response to SHW migration ^(78)^. Beyond an understanding of the scope of skilled health migration governance in Nigeria, our framework provides multiple entry points and interconnections useful for understanding how SHW migration is handled in under governed settings. It thus offers insights for designing future policy interventions and evaluating their utility for achieving health workforce sustainability in Nigeria and other national settings facing similar challenges.

**3. Update no.s of emigrant Nigerian health workers working overseas.**

This has been updated on page 3, line 67 – 71 as follows:

“Between 2008 and 2021, a total of 36,467 Nigerian doctors migrated to the United Kingdom. There was a steady increase from 1,798 that migrated in 2008 to 4,880 in 2021. A larger trend was observed for nurses. Between 2002 and 2021, a total of 60,729 Nigerian nurses had migrated to the United Kingdom. There was a steady increase from 1,393 nurses that migrated in 2002, to 5,543 in 2021^(4)^.

**4. Clarify whether any of the sample were return migrants.**

We did not capture data for return migrants. Our inclusion criteria were any skilled health worker that resided in Nigeria irrespective of the duration and their migration status.

**5. Update discussion/conclusions to reflect these changes requested by this and the other reviewer.**

We have reflected the changes in the discussion as recommended.

**References**

1.	Abimbola S, Negin J, Martiniuk AL, Jan S. Institutional analysis of health system governance. Health Policy Plan. 2017;32(9):1337-44.10.1093/heapol/czx083

2.	Bevir M. Governance and Interpretation: What are the Implications of Postfoundationalism? Public Administration. 2004;82(3):605-25

3.	Chudnovsky M, Trujillo NE. Conflict between Formal and Informal Rules in Policy Coordination : The Case of Child Homelessness in Mexico City. Latin American Policy. 2019;10(1):120-43

4.	Walton-Roberts M, Runnels V, Rajan SI, Sood A, Nair S, Thomas P, et al. Causes, consequences, and policy responses to the migration of health workers: key findings from India. Human Resources for Health. 2017;15:1-18.10.1186/s12960-017-0199-y

5.	Labonte R, Sanders D, Mathole T, Crush J, Chikanda A, Dambisya Y, et al. Health worker migration from South Africa: causes, consequences and policy responses. Hum Resour Health. 2015;13:92.10.1186/s12960-015-0093-4
